# The Psychometric Properties of the Swedish Version of the General Self-Efficacy Scale: A Rasch Analysis Based on Adolescent Data

**DOI:** 10.1007/s12144-016-9551-y

**Published:** 2017-01-11

**Authors:** Victoria Lönnfjord, Curt Hagquist

**Affiliations:** Centre for Research on Child and Adolescent Mental Health, Karlstad University, Sweden, SE-651 88 Karlstad, Sweden

**Keywords:** Self-efficacy, Adolescents, Rasch analysis, Psychometric, Sweden, General Self-Efficacy Scale

## Abstract

Self-efficacy describes people’s belief in their own ability to perform the behaviors required to produce a desired outcome. The purpose of this study was to examine the psychometric properties of the Swedish version of the General Self-Efficacy Scale (GSES) with an adolescent sample, using Rasch analysis. The scale was examined with a focus on invariant functioning along the latent trait as well as across sample groups. The data were collected 2009 and 2010 among 3764 students aged between 13 and 15 years, in the 7^th^ to 9^th^ grade, in compulsory schools in the municipality of Karlstad, Sweden. The item fit was acceptable, the categorization of the items worked well and the scale worked invariantly between years of investigations. Although the GSES worked well as a whole, there was some evidence of misfit indicating room for improvements. The targeting may be improved by adding more questions of medium difficulty. Also, further attention needs to be paid to the dimensionality of the GSES as well as to whether the psychometric properties of GSES are affected by using more recent data.

## Introduction

The concept of self-efficacy was developed by Albert Bandura in his social cognitive theory. Self-efficacy is defined as people’s belief in their ability to perform the behaviors required to produce a desired outcome (Bandura 1977). The concept of self-efficacy has been used in several research areas, such as educational research (see Schunk 1991; Silver et al. 2001; Usher and Pajares 2008; Zimmerman 2000), organizational research (see Chen et al. 2001), and social work research (see Jackson and Huang 2000; Ramo et al. 2010; Schmall 1994).

There are four sources of information that impact a person’s self-efficacy: performance accomplishment, vicarious experience, verbal persuasion, and physiological states (Bandura 1977). According to Bandura (1993, 1997), self-efficacy affect how people feel, think, motivate themselves, and behave. It is hypothesized that people with low self-efficacy for accomplishing a task may refrain from performing the task at hand (Schunk 1991; Bandura 1997). Low self-efficacy then becomes a vicious circle: *“Lack of faith in ability produces lack of action. Lack of action contributes to more self-doubt. They become doubtful of their own capabilities and are more easily stressed and more frequently depressed than people with high self-efficacy”* (Singh and Udainiya 2009). People with high self-efficacy for accomplishing a task should readily attempt the task. Bandura (1977) also stated that these people work harder, and are more persistent when difficulties arise, than people with low self-efficacy. Self-efficacy differs conceptually from related motivational constructs, such as outcome expectations (Zimmerman 2000), perceived control (Endler et al. 2001), self-concept, or locus of control (Zimmerman 2000; Pastorelli et al. 2001).

The theory originally stated that self-efficacy is situation-specific (Bandura 1977), meaning that a person could experience high self-efficacy in one situation and low in another. As a result, a large number of domain-specific instruments measuring self-efficacy have been developed, for example, a career decision-making self-efficacy scale (Betz et al. 1996), a nursing self-efficacy scale (Hagquist et al. 2009), and an alcohol abstinence self-efficacy scale (DiClemente et al. 1994). However, other researchers have proposed that self-efficacy may be generalized (Schwarzer and Jerusalem 1995; Sherer et al. 1982; Eden 1988). As the most important source of information that contributes to a person’s self-efficacy is performance accomplishment, it has been argued that an individual’s experience of failure or success in different situations should result in a generalized type of self-efficacy (Sherer et al. 1982). Even though Bandura (1997) was against an ‘”all-purpose measure”, he acknowledged that self-efficacy can be generalized when commonalities are cognitively structured across activities, for example, when tasks require similar subskills or when the skills required to accomplish dissimilar activities are acquired together. Bandura (1997) also described “transforming experiences” that can strengthen a person’s beliefs in other areas than where the success was achieved. Several scales to measure general self-efficacy exist within research; one was developed by Schwarzer and Jerusalem (1995) and another by Sherer and colleagues (1982), which in turn has a short version that was developed by Chen and colleagues (Chen et al. 2001).

The General Self-Efficacy Scale (GSES), developed by Schwarzer and Jerusalem (1995), has been translated into 31 different languages (see http://userpage.fu-berlin.de/health/selfscal.htm). The scale has been psychometrically tested in different populations and in different cultures (e.g Scholz et al. 2002; Luszczynska et al. 2005). Results show it to be a reliable, valid, and unidimensional scale. However, most research testing the psychometric properties of the scale have been based on theories within the classical test theory paradigm; only two studies have evaluated the psychometric properties of the GSES using Rasch analysis, one with a sample of adults with spinal cord injury (Peter et al. 2014) and the other with morbidly obese adults (Bonsaksen et al. 2013). Thus, neither of these studies used samples of adolescents. The scale was in fact intended to be used with adolescents as well as the general adult population (Schwarzer and Jerusalem 1995). The way in which adolescents develop and exercise their self-efficacy during this transitional period can play a key role in setting the course their life paths take (Bandura 2006). Studies have shown that an adolescent’s self-efficacy affects their physical activity (Feltz and Magyar 2006), their risk-taking behavior, and their health decisions (Schwarzer and Luszczynska 2006). The Swedish version of the GSES (Koskinen-Hagman et al. 1999) has been psychometrically evaluated based on classical test theory. The sample consisted of people from the general population and people on sick leave, aged 19 to 64 years. Results showed high internal consistency (α = 0.90), unidimensionality, and factor loadings ranging between 0.64 and 0.80 (Löve et al. 2012).

The purpose of the present study was to examine the psychometric properties of the Swedish version of the GSES with an adolescent sample. Since invariant comparisons of general self-efficacy between different samples are essential, we used the Rasch model, which has invariance as an integral property. The Rasch model has not previously been applied on general self-efficacy adolescent data.

## Methods

### Material

The data for this study were collected as part of a school prevention project in collaboration between the municipality of Karlstad in Sweden and the Centre for Research on Child and Adolescent Mental Health (CFBUPH) at Karlstad University, Sweden. Data were collected about social relations, classroom climate, bullying, and mental health. The overall aim of the project was to promote good mental health among children and adolescents.

### Data Collection

The data in the present study were collected 2009 and 2010 among students aged between 13 and 15 years in the 7^th^ to 9^th^ grades in compulsory schools in the municipality of Karlstad. The data collection was carried out by a research team at CFBUPH. All students received both written and oral information about the aim of the study, that their participation was voluntary, and that they had the right to withdraw their participation at any time. Due to the age of the children in the 7^th^ and 8^th^ grades, written information was given to the parents, and those who did not want their children to participate were asked to notify the grade teacher. Eight out of nine compulsory schools participated in the data collection in 2009. The questionnaire consisted of 109 questions, with the items about general self-efficacy as number 58. The questionnaire for 2010 consisted of 116 questions, with the items about general self-efficacy as number 63. By 2010, one of the compulsory schools in the municipality had closed and that year all eight of the remaining compulsory schools participated. Table 1 shows the number of participants and non-participants for 2009 and 2010.

**Table 1:** Information about participants in 2009 and 2010

Year	Number of students		Number of completed questionnaires		Non-participants n (%)	
	2009	2010	2009	2010	2009	2010
Grade 7						
Total	655	707	578	656	76 (11.6)	51 (7.2)
Boys	330	348	291	322	39 (10.6)	26 (7.5)
Girls	325	359	287	331	38 (10.8)	28 (7.8)
Grade 8						
Total	732	711	590	636	142 (19.4)	75 (10.5)
Boys	358	349	280	304	78 (19.6)	45 (12.9)
Girls	374	362	310	328	64 (16.0)	34 (9.4)
Grade 9						
Total	753	802	578	712	175 (23.8)	90 (11.2)
Boys	383	386	297	325	65 (20.7)	61 (15.8)
Girls	370	416	282	382	73 (24.2)	34 (8.2)
Total	2140	2220	1760	2004	367 (17.1)	216 (9.7)
Boys	1071	1083	868	953	203 (19.0)	132 (12.2)
Girls	1069	1136	879	1046	109 (10.2)	95 (8.4)

^a^ Includes students from all but one compulsory school in the municipality of Karlstad in Sweden

Table 1 show that the proportion of the non-participants decreased from 17.1% for 2009 to 9.7% for 2010. In both years, a higher proportion of non-participants were found in the higher grades.

### Instrument

The GSES consists of ten items, see Appendix Table 6. The responses to the items are summarized across respondents, yielding a score between 10 and 40; higher scores indicate higher self-efficacy. The Swedish version of the GSES is the instrument investigated in this study. The GSES was translated into Swedish in 1999 (Koskinen-Hagman et al. 1999) and the adaptation followed a group consensus model (personal communication with Koskinen-Hagman Dec 5, 2015). Although, the process of translation into Swedish has not been reported in any papers, the general principles for the translation process have previously been described, as part of a study investigating general self-efficacy as a universal construct, in the following way: *“The procedure included back translations and group discussions. Since the goal was to achieve cultural-sensitive adaptations of the construct rather than mere literal translations, the translators acquired a thorough understanding of the general self-efficacy construct”* (Scholz et al. 2002)*.* This description corresponds to the group consensus model described by the Swedish translator.

### Analysis Using the Rasch Model

Rasch analysis can be used to examine whether responses to individual items can be combined into a unidimensional composite measure, enabling us to distinguish individuals at the high and low levels of the latent trait (Andrich 1988). The Rasch model estimates item and person parameters independently of each other and places both parameter estimates on the same latent variable, which offers opportunities to examine the targeting, in other words, the locations of the items relative to the respondents. If the targeting is bad, the reliability will be lower, which makes it hard to differentiate people along the latent trait

Since invariance is an integral property of the Rasch model, a test of fit between the data and the model is a test of whether the instrument works invariantly or not. In the Rasch analysis, the focus is on the operating characteristics of the items along the whole continuum of a latent trait, not on a single summary measure. Expected value curve (EVC), sometimes called Item Characteristic Curves, are useful graphical tools for checking the fit of the data to the Rasch model, complementing formal test statistics. The EVC predicts the responses to the items as a function of the items and the respondent’s locations on the latent trait. These expected values are compared with the observed values. If an item shows differential item functioning (DIF), more than one EVC is required to predict the responses to that item. This means that members of one group score differently on an item than members of another group, given the same location on the latent trait. In DIF analysis of an item set, several items may show evidence of DIF, consisting of real DIF items as well as artificial DIF items (Andrich and Hagquist 2012, 2015). Real DIF is inherent to an item and affects the person measures, while artificial DIF does not, because it is an artifact of the procedure to identify DIF. Since real DIF affects the person measurement and the comparisons between groups, different options to address real DIF may be considered. One option is to simply remove the real DIF item(s); another option is to take the DIF into account based on principles of equating. While the first option will decrease the reliability and person separation, the second will not have any effect on the reliability and person separation. Therefore, it is usually preferable to resolve an item instead of removing it. Given that resolving an item, like removing an item, may affect the validity, from that perspective resolving DIF is only justified if the source of DIF can be shown to arise from some source irrelevant to the variable being assessed and therefore deemed dispensable.

The data may statistically fit the polytomous Rasch model (Andrich 1978) although the response categories do not operate in the intended order. Such threats to measurement may also be detected by the Rasch model thanks to its sensitivity to the categorization of the items. Hence, the Rasch analysis may facilitate decisions about the number of response categories that would be optimal as well as the phrasing of the categories.

In the present study, the following issues were analyzed:

- Person separation
- Whether items cover the full range of ability levels of the latent trait i.e. targeting
- Whether there is any threshold disordering
- Item fit
- Differential item functioning (DIF) for gender, age, and grade
- Local dependency

The procedures for analyzing theses aspects are described below. The analyses were conducted using the software program RUMM 2030 (Andrich et al. 2012).

- The person separation index (PSI) is the measure of reliability employed in this study; it is analogous with Cronbach’s alpha when the data is normally distributed (Pallant and Tennant 2007).
- Targeting for GSES was analyzed in the present study by examining the person–item threshold distribution (Tennant and Conaghan 2007).
- Disordered thresholds occur when respondents cannot discriminate between the response options. This could reflect the phrasing of the response, or it could be misinterpreted or confusing, or there could be too many response options (Pallant and Tennant 2007). The ordering of the thresholds is therefore important.
- The item fit can be examined in different ways: graphically with the EVC and formally with fit residuals and chi-square tests (Hagquist et al. 2009). A value between -2.5 and 2.5 is considered acceptable for the fit residuals (Pallant and Tennant 2007). The reported p-values are chi-square statistics based on a comparison between the observed means and the expected values (Hagquist 2001) in equal-sized class intervals of people (i.e. groups representing different ability levels) along the latent trait. One thing to bear in mind is that a large sample size always gives low p-values. With a large sample size, the parameters are estimated with great precision, and any misfit will be exposed (Andrich 1988). Therefore, even if there are significant p-values indicating that the expected values and the model do not fit, the items may be retained. To reconcile the sensitivity of the formal test but still have the advantage of the graphical representation, Andrich and Styles (2010) suggest adjusting the sample size. The chi-square statistic is proportional to the sample size, and what happens in practice according to Andrich and Styles (2010) is that the chi-square statistic is multiplied by the new sample size divided by N. For this reason, formal tests with an adjusted sample size (n = 900) were performed in the present study.
- In order to make invariant comparisons, a measurement instrument has to function the same way along the latent trait and across the groups that are to be compared, for example between girls and boys, across age groups, across sampling years, between cultures, and across countries (Andrich 1988; Hagquist et al. 2009). Lack of invariance across groups is referred to as DIF. We analyzed DIF graphically by the EVCs and formally by analysis of variance (ANOVA). There are two types of DIF: uniform and non-uniform. Uniform DIF is when the EVCs for each group are parallel, and non-uniform DIF means that the EVCs are non-parallel (Andrich and Hagquist 2012). The groups of interest (person factors) in this analysis are: *grade* (7^th^, 8^th^ or 9^th^), *year* (2009 or 2010) and *gender* (girl or boy). The ANOVA of standardized residuals, shows main effects for class interval and a main effect for person factors (uniform DIF), as well as an interaction effect between class interval and person factors (non-uniform DIF). To distinguish between real and artificial DIF, the item with the highest F-value must be identified. Andrich and Hagquist (2012) suggest that you should resolve the item with the highest F-value. Resolving an item means that an item is split into the specific sample groups, for example, *gender* will result in one item for girls and one item for boys, in which the values for the excluded group are treated as missing. In the present study, items were resolved for DIF starting with the worst-fitting item; thereafter a new ANOVA was performed to identify whether any additional items needed to be resolved.
- Local independence refers to the idea that for the same value of β (person parameter, that is, a person’s ability), there is no further relationship between responses to any pair of items (Marais and Andrich 2008). Correlations between residuals may indicate local independence. There are two different types of local dependency: one is *response dependency* (Marais and Andrich 2008). It means that the response that a person gives to one item depends on the response that the same person gave to a previous item. Evidence of response dependency was analyzed in the present study by identifying items with residual correlations above 0.3 in the Person–item residual correlation matrix. The second type of local dependency is *violation of unidimensionality*, or what Marais and Andrich (2008) refer to as trait dependence, which reflects the presence of more than one trait. Unidimensionality is an important aspect of construct validity. Evidence of multidimensionality was analyzed in the present study by first identifying positive or negative principal component loadings and then conducting t-tests of differences in person–location values generated from these two subsets of items. Although trait and response dependence are conceptually different, they are hard to distinguish, both empirically and in the literature (Marais and Andrich 2008).

## Results

### Original Item Set (10 items)

Power of analysis of fit was “*Excellent”* on all tests conducted. This indicates that the people are spread out throughout the continuum, and not clustered around the same location.

#### Reliability, Targeting, and Threshold Ordering

The PSI value for all ten items was 0.8998 (Cronbach’s α 0.93788) with extremes and 0.8737 (Cronbach’s α 0.90001) without extremes.

The person–item threshold distribution showed that the mean location was 1.411 with a standard deviation of 2.241. It also showed that there were a lot of easier items but there were no items covering the range from 0.5 to 1.5 logits. This was also where most people were located; i.e. there were no items targeting these people. Furthermore there were extreme values at both ends. There were no questions covering locations over 4.25 logits or under -3.25 logits. So no items targeted these people either. The different means for the person factor subgroups differ between -0.271 and +0.242 from the mean of the whole sample. The means for the person factor *grade* were 1.497 for 7^th^ grade, 1.394 for 8^th^ grade, and 1.356 for 9^th^ grade, means for the person factor *year* were 1.397 for 2009 and 1.424 for 2010, and means for the person factor *gender* were 1.169 for girls and 1.685 for boys. This implies that out of all the subgroups, the instrument was targeted best for girls (1.169). Also, these values were interpreted as showing that the boys as a group have higher self-efficacy.

The analysis showed no disordered thresholds, which indicates that the response format works well.

#### Item Fit – Test of Invariance at a General Level

**Fig. 1:**
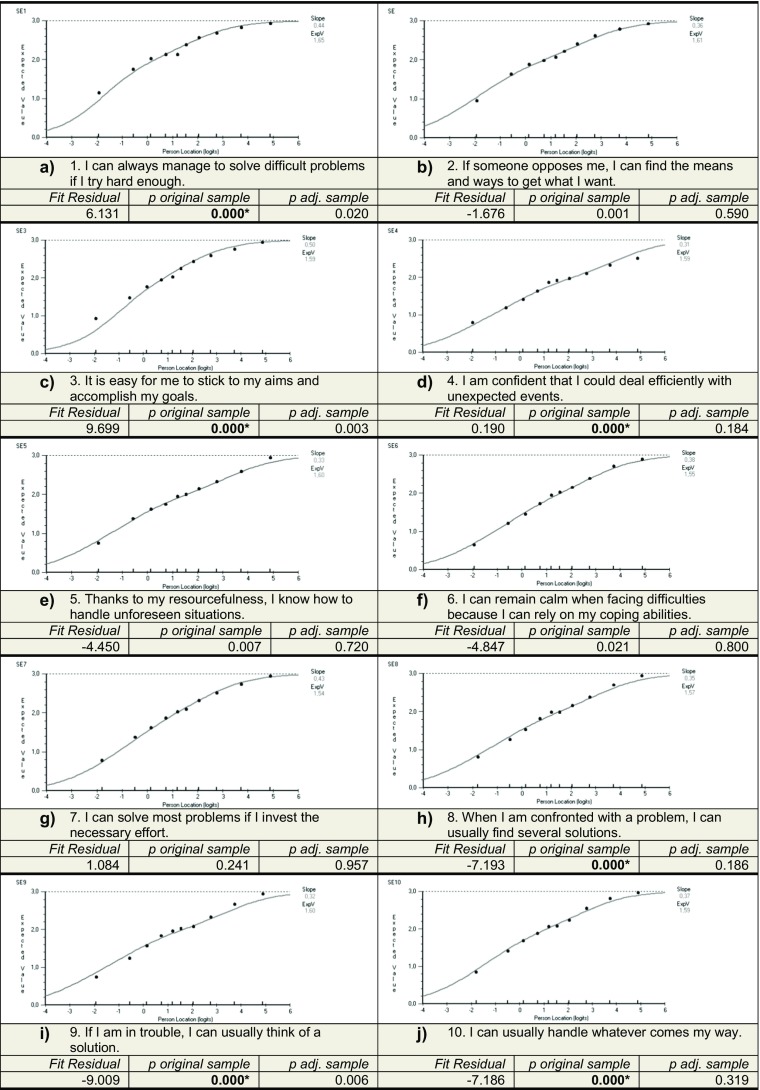
Fit residuals, p-values for the chi-square tests (*n* = 3272), with significant p-values in bold, and p-values for the chi-square tests with an adjusted sample size (n = 900), both chi-square tests with Bonferroni adjustment (0.001). Also in the figure are the EVCs for all ten items

Figure 1 shows the EVCs, along with results from formal tests of item fit.

Figure 1 shows that two items (1 and 3) were under-discriminating and five items (5, 6, 8, 9, and 10) were over-discriminating. Negative residuals indicate over-discrimination and positive residuals indicate under-discrimination. The p-values for the items are less than 0.001 (Bonferroni adjustment) for all the items except items 2, 5, 6, and 7. To reconcile the sensitivity of the formal test due to the large sample size, the sample size was adjusted, in this case to 900; the p-values for the formal chi-square test with the adjusted sample size can also be seen in Fig. 1. Using this adjusted sample size, no items show evidence of statistical misfit.

Figure 1 g shows item 7, which has a good fit in both the graphical and formal investigation. Figure 1c shows item 3, an item that shows under-discrimination according to the fit residual. This indicates that students with low self-efficacy tend to score too high on this particular item and students with high self-efficacy tend to score too low, according to the Rasch model. Figure 1e shows item 5, an item that shows over-discrimination according to the fit residual. The opposite pattern occurs here, this indicates that students with low self-efficacy tend to score too low on this particular item and students with high self-efficacy tend to score too high, according to the Rasch model.

Even though the graphical representations in Fig. 1c and e show a little over- and under-discrimination, the deviation from the EVC is minor. These minor deviations also apply for the EVC curves for the rest of the items shown in Fig. 1.

Table 2 shows the item location for each item, and the spread of the item location values corresponds to the severity of each item. Item 4 represents the most difficult item whereas item 1 represents the easiest. Item 5 and 9 have almost the same item location.

**Table 2:** Item location for all ten items

Item	Location
1. I can always manage to solve difficult problems if I try hard enough	–0,618
2. If someone opposes me, I can find the means and ways to get what I want	–0,527
3. It is easy for me to stick to my aims and accomplish my goals	–0,083
4. I am confident that I could deal efficiently with unexpected events	0,626
5. Thanks to my resourcefulness, I know how to handle unforeseen situations	0,146
6. I can remain calm when facing difficulties because I can rely on my coping abilities	0,275
7. I can solve most problems if I invest the necessary effort	0,038
8. When I am confronted with a problem, I can usually find several solutions	0,133
9. If I am in trouble, I can usually think of a solution	0,141
10. I can usually handle whatever comes my way	–0,130

#### Differential Item Functioning – Test of Invariance at a Finer Level

The groups of interest (person factors) in this analysis were: *grade* (7^th^, 8^th^, or 9^th^), *year* (2009 or 2010), and *gender* (girl or boy). Table 3 shows the results from the analysis concerning the person factor *gender*. The analysis showed no non-uniform DIF for that person factor. Table 3 also shows that for the person factor *gender,* uniform DIF was found in items 1, 2, 3, 4, and 6 when the sample size was intact. After the sample size was adjusted, only the DIF in item 6 remained statistically significant.

**Table 3:** Results from analyses detecting DIF for the person factor, *gender* in the GSES with the whole sample (n = 3272) and adjusted sample size of 900

Item	DIF – Gender (original set)Original sample size			DIF – Gender (original set)Adjusted sample size		
	*Class interval* F (p)	*Gender* F (p)	*CI by Gender* F (p)	*Class interval* F (p)	*Gender* F (p)	*CI by Gender* F (p)
1. I can always manage to solve difficult problems if I try hard enough	8.07525(0.000000*)	13.17557(0.000321*)	1.98541(0.037054)	2.18321(0.021210)	3.56212(0.059435)	0.53677(0.848287)
2. If someone opposes me, I can find the means and ways to get what I want	3.48655(0.000243*)	12.61057(0.000449*)	1.18846(0.297540)	0.94322(0.486418)	3.41156(0.065095)	0.32152(0.968111)
3. It is easy for me to stick to my aims and accomplish my goals	8.73036(0.000031*)	15.21365(0.000139*)	2.26552(0.015906)	2.36150(0.012293)	4.11518(0.042816)	0.61281(0.786832)
4. I am confident that I could deal efficiently with unexpected events	5.55753(0.000065*)	15.86338(0.000094*)	0.13405(0.998791)	1.50454(0.141655)	4.29456(0.038538)	0.03629(0.999995)
5. Thanks to my resourcefulness, I know how to handle unforeseen situations	3.28658(0.000499*)	0.47466(0.490938)	0.93225(0.495523)	0.88891(0.534590)	0.12838(0.720214)	0.25214(0.986324)
6. I can remain calm when facing difficulties because I can rely on my coping abilities	2.73061(0.003550)	81.51663(0.000019*)	0.28310(0.979519)	0.73931(0.672980)	22.07059(0.000008*)	0.07665(0.999878)
7. I can solve most problems if I invest the necessary effort	1.43065(0.168879)	6.63717(0.009964)	0.76013(0.653637)	0.38696(0.941684)	1.79522(0.180630)	0.20560(0.993546)
8. When I am confronted with a problem, I can usually find several solutions	6.64186(0.000000*)	0.19805(0.656380)	2.01034(0.034504)	1.79910(0.064698)	0.05365(0.816904)	0.54455(0.842349)
9. If I am in trouble, I can usually think of a solution	13.84965(0.000073*)	7.81948(0.005234)	0.75172(0.661498)	3.76028(0.000134*)	2.12304(0.145452)	0.20410(0.993722)
10. I can usually handle whatever comes my way	6.00426 (0.000000*)	0.00034 (0.984514)	2.03790 (0.031790)	1.62288 (0.104311)	0.00009 (0.991952)	0.55082 (0.837498)

* = Significance level below Bonferroni probability adjustment of 0.001667 (N = 30)

Analyses showed (not shown in the table) that uniform DIF was found in items 1, 5, and 9, for the person factor *grade.* When the sample size was adjusted, DIF found in those items was no longer statistically significant. Analyses also showed (not shown in the table) that there was no uniform or non-uniform DIF for the person factor *year,* irrespective of sample size, which indicates that the instrument works invariantly between years.

#### Local Independence

Looking at the residual correlation matrix, no correlations above a value of 0.3 were found, which indicates that there is no evidence, or only minor evidence, of local independence in the form of response dependence.

Investigations of PC loadings revealed that there might be a violation of unidimensionality, hence trait dependence. The loadings suggest that item 1, 2, and 3 represented one dimension and items 4, 5, 6, 7, 8, 9, and 10 represented another dimension.

To investigate this further, a paired t-test was conducted, where one set contained items 1, 2, and 3, which had a positive component–item residual, and the second set contained items 4, 5, 6, 7, 8, 9, and 10, which had a negative component–item residual. Analyses showed that the person location values from the two subsets were significantly different for 8.38%, thus exceeded the critical value of 5%, which may indicate trait dependence.

### Revised Item Set (11 Items)

The following results describe the instrument after item 6 (*I can remain calm when facing difficulties because I can rely on my coping abilities*) was resolved for gender. To resolve an item in this case refers to splitting item 6 into two items, one for girls and one for boys, so this set includes 11 items.

#### Differential Item Functioning

To distinguish between real and artificial DIF, the item with the highest F-value was identified. This meant that item 6 for the person factor *gender* was resolved. Table 3 shows that the F-value for item 6 was 81.51663.

**Fig. 2:**
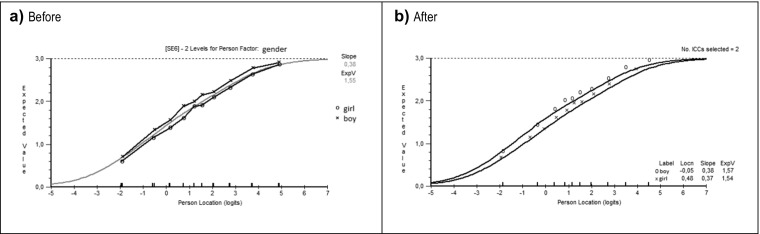
EVC for item 6, *I can remain calm when facing difficulties because I can rely on my coping abilities*. **a**) EVC before resolving the item (the line with x represents the boys and the line with o represents the girls); **b**) EVC after the item was resolved (here, the signs are reversed, so o represents boys and x represents girls (*n* = 3272))

Figure 2 displays a graphical representation of a uniform DIF for item 6, for the person factor *gender*. The curves for boys and girls deviated in a parallel way, as seen in Fig. 2a. Irrespective of a boy’s or girl’s ability, the boys tended to answer higher on this item than girls, given the same location on the latent trait. The same pattern appeared for item 4. The opposite pattern occurred that girls tended to answer “higher” than boys on items 1, 2, and 3.

After item 6 was resolved, no DIF was statistically significant in either the person factor *gender* or *grade* when the sample size was adjusted, as shown in Table 4.

**Table 4:** Results from analyses detecting DIF for the person factors *gender, grade*, and *year* in the revised set of the GSES with adjusted sample size of 900, after item 6 was resolved for *gender*

Item	DIF – Gender (revised set)			DIF – Grade (revised set)			DIF – Year (revised set)		
	*Class interval* F (p)	*Gender* F (p)	*CI by Gender* F (p)	*Class interval* F (p)	*Grade* F (p)	*CI by Grade* F (p)	*Class interval* F (p)	*Year* F (p)	*CI by Year* F (p)
1	1.82039 (0.060951)	2.00291 (0.157361)	0.77458 (0.640106)	1.76603 (0.070872)	2.98711 (0.050948)	0.15212 (0.999986)	1.81320 (0.062169)	0.00562 (0.940157)	0.27549 (0.981263)
2	1.10164 (0.358785)	1.79524 (0.180635)	0.27832 (0.980577)	1.08139 (0.373869)	0.6779 (0.507963)	0.31272 (0.997383)	1.07483 (0.378815)	0.09952 (0.752462)	0.24482 (0.987707)
3	2.46777 (0.008810)	2.33724 (0.126680)	0.42935 (0.919686)	2.44316 (0.009541)	0.78735 (0.455376)	0.44692 (0.977394)	2.44730 (0.009404)	0.18641 (0.66601)	0.11635 (0.999311)
4	1.23895 (0.267307)	6.59131 (0.010415)	0.16158 (0.97440)	1.22747 (0.274254)	0.43657 (0.646386)	0.34260 (0.995307)	1.21708 (0.280607)	1.97774 (0.16000)	0.04981 (0.999981)
5	0.67249 (0.734326)	0.79226 (0.373666)	0.29935 (0.974985)	0.65795 (0.747375)	2.95460 (0.052625)	0.31933 (0.997002)	0.67851 (0.728887)	0.06055 (0805714)	0.26867 (0.982848)
7	0.31422 (0.970485)	0.62748 (0.428518)	0.27775 (0.980713)	0.32235 (0.967827)	0.74906 (0.473130)	0.28833 (0.998469)	0.32332 (0.967503)	0.58575 (0.444266)	0.55768 (0.832139)
8	1.71143 (0.082304)	0.64407 (0.422473)	0.80334 (0.613277)	1.70967 (0.082714)	1.05015 (0.350343)	0.22769 (0.999703)	1.69367 (0.086335)	0.00560 (0.940362)	0.23159 (0.989959)
9	3.84241 (0.000102^a^)	4.10567 (0.043056)	0.16237 (0.997391)	3.74702 (0.000120^b^)	1.82519 (0.161821)	0.20476 (0.999862)	3.83213 (0.000107^c^)	0.10373 (0.747497)	0.24958 (0.986818)
10	1.29485 (0.235451)	0.33113 (0.565133)	0.38035 (0.944771)	1.27094 (0.248722)	0.21111 (0.809725)	0.52130 (0.949211)	1.29953 (0.232909)	0.13959 (0.708778)	0.25319 (0.986118)
6 girl	0.30156 (0.974168)	0.00000 (0.999999)	0.00000 (0.999999)	0.29568 (0.975839)	0.26627 (0.766345)	0.35183 (0.994287)	0.29879 (0.974964)	0.50467 (0.477821)	0.57274 (0.819714)
6 boy	0.59215 (0.803651)	0.00000 (0.999999)	0.00000 (0.999999)	0.53485 (0.849046)	0.28964 (0.748699)	0.29943 (0.997924)	0.58101 (0.812793)	0.00089 (0.976130)	0.30391 (0.973368)

^a^ = Significance level below Bonferroni probability adjustment of 0.001724 (*N* = 29)

^b^ = Significance level below Bonferroni probability adjustment of 0.001515 (*N* = 33)

^c^ = Significance level below Bonferroni probability adjustment of 0.001852 (*N* = 27)

#### Targeting

**Fig. 3:**
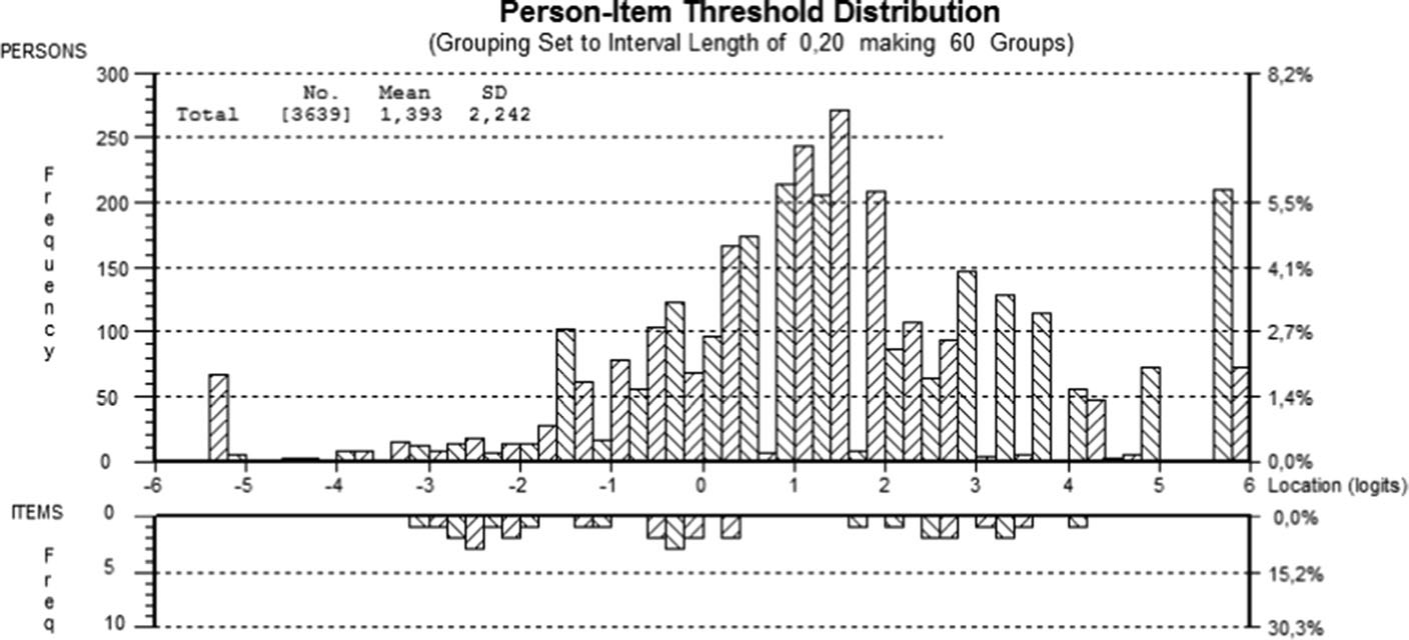
The Person–item threshold distribution for all participants (*n* = 3639)

Figure 3 shows the person–item threshold distribution. The number of people was 3639, and the mean location was 1.393, with a standard deviation of 2.242.

The targeting of the instrument when item 6 was resolved compared to when the item set was intact does not differ significantly. The revised set had a slightly lower mean person location. There were a lot of easier items but there were no items to cover the range from 0.5 to 1.5 logits. This was also where the figure shows the most people; i.e. there were no items targeting these people. Furthermore there were extreme values at both ends. There were no questions to cover location values over 4.25 logits or lower than -3.25 logits. So no items target these people either.

#### Threshold Ordering

The analysis showed no disordered thresholds, which indicates that the response format works well.

### Comparison of Person Measures

To examine the effect of the DIF items on person measurement, the mean values of the person–item threshold distribution for boys and girls were compared before and after the DIF for the person factor *gender* was resolved. The results can be seen in Table 5. Also included in the analysis and shown in Table 5 are the person measures if item 6 is removed.

**Table 5:** Mean values of the person–item threshold distribution for the GSES before and after item 6 is resolved for the person factor *gender* and when item 6 is removed

	Boys, mean (n 1726)	Girls, mean (n 1891)	Boys - Girls, mean	PSI
All items intact	1.685^a^ (SD 2.44)	1.169^b^ (SD 2.00)	0.516	0.89983
1 DIF resolved	1.636^c^ (SD 2.44)	1.179^d^ (SD 2.01)	0.457	0.89963
Item 6 removed	1,665^e^ (SD 2,40)	1,209^f^ (SD 1,98)	0,456	0.89012

^a-b=^ difference between means before any items are resolved

^c-d=^ difference between means after item 6 is resolved

^e-f=^ difference between means when item 6 is removed

Table 4 shows that the differences between the mean value for boys and girls in the original set, before any item was resolved, was 0.516, and the difference between the mean value for boys and girls in the revised 11-item set, after item 6 was resolved, was 0.457. Given that artificial DIF never affects the person measure, this confirms that the DIF was real. Table 4 also shows that the PSI value is slightly higher if item 6 is resolved rather than removed from the item set.

## Discussion

The purpose of this study was to examine the psychometric properties of the Swedish version of the GSES with an adolescent sample, using Rasch analysis. The analysis of the fit residual for the GSES shows seven items that are over- or under-discriminating, of which five show statistically significant misfit. Given the large sample size and the fact that even the smallest deviation is detected, which in turn will result in fit residuals, the graphical investigation of the EVC is important to judge the magnitude of the misfit. In the items that show misfit according to the formal test statistics, the observations are located close to the EVC, indicating that the misfit might be only minor. This hypothesis is confirmed when an adjustment of the sample size is used as a heuristic tool for the evaluation of an instrument. When the sample size is adjusted, the DIF is no longer statistically significant for any of the items for *grade* and only one item remains statistically significant for *gender*. But after that item is resolved, no more DIF is found when the sample size is adjusted. It is important to bear in mind that, when adjusting the sample size, the parameters are estimated with good precision but have less power to detect misfit (Bergh 2014).

There are a few results that are quite similar between the original 10-item set and the revised 11-item set. The high PSI values, 0.8998 for the original set and 0.89963 for the revised set, indicate high reliability for the GSES. As expected, the PSI is slightly higher if item 6 is resolved rather than removed. In this study, the mean location is 1.411 in the original set and 1.393 in the revised set, indicating that the population has higher self-efficacy than the instrument is supposed to capture. The person–item distribution also shows that there are no questions covering the range where the largest proportion of people is located, so one improvement that can be made to the instrument is to add items with medium difficulty. This interpretation applies in both the original and the revised set. Another result shared by the two item sets concerns the response format, where the four qualitative response options (*Not true at all*; *Hardly true*; *Moderately true*; *Exactly true*) seem to work well.

The results presented may be discussed in relation to previous concerns about the GSES and also in relation to theoretical assumptions. The results reveal that there might be a violation of unidimensionality; it might be a trait dependence in the original 10-item set of the GSES, in so far that items 1, 2, and 3 represent one dimension and items 4, 5, 6, 7, 8, 9, and 10 represent another. The former group contains aspects of how much a person perseveres. The latter group of items covers the aspect of self-efficacy that relates to new, surprising or unexpected situations. It seems unlikely that the questions cover different dimensionalities, but rather that items in the latter group are phrased more similarly. Indeed, trait dependence is also found in items that are linked by attributes such as common stimulus materials, common item stems, common item structures, or common item content (Marais and Andrich 2008). Other research (Zhou 2015) has questioned the unidimensionality of the 10-item version of GSES, talking about *action self-efficacy* (items 1, 6, 7, 8, and 9), meaning self-efficacy in a pre-intentional phase, and *coping self-efficacy* (items 2, 3, 4, 5, and 10), meaning self-efficacy in a post-intentional phase. Further analysis (not shown in the paper) with five different samples (7^th^ grade, 8^th^ grade, 9^th^ grade, girls and boys) all shows evidence of items 1, 2, and 3 belonging to one dimension. All but one sample have had positive PC-loadings; the 8^th^ grade sample is the one sample where items 1, 2, and 3 have negative PC loadings. What could these results imply for the usage of the instrument?

There may also be some concerns about the phrasing of some items. In particular, item 6 seems to be problematic. The phrasing of that statement in Swedish is a bit ambiguous. The back translation is, *“Because of my own ability, I feel calm even when I am facing difficulties”,* which does not explicitly specify coping, as in the English version (“*I can remain calm when facing difficulties because I can rely on my coping abilities.*”) There are a few aspects to consider when answering item 6. But this is also the only item that directly taps information about the aspect of physiological state that could impact a person’s self-efficacy beliefs according to Bandura (1977). Another item that might be a cause for concern regarding the translation is item 7, which does not capture a person’s belief about their *own* internal resources as the English version does. The back translation is, *“Whatever happens, I’ll always manage”* (compared to the English version, ”*I can solve most problems if I invest the necessary effort.”)* A person could interpret the Swedish item 7 in terms of external resources, for example, having supportive family or friends, enough money, or a place to live. Analysis, when item 7 is removed (not included in the paper) was performed showing results that did not considerably alter the outcomes from the psychometric evaluation of the GSES, indicating that item 7 was probably read in the context of the other items. In other words, the students interpreted item 7 to be about internal resources. Other researchers (Bonsaksen et al. 2013) have also discussed the content of item 2 (“*If someone opposes me, I can find the means and ways to get what I want”*) as problematic, as it is the only item to include an interpersonal aspect, both in the English and translated versions (Norwegian and Swedish). But this item might reflect another one of the sources of information that impact a person’s self-efficacy according to Bandura (1977), namely, verbal persuasion. If someone *opposes* you, this could mean that the person may perform some hostile actions or it could be in the form of discouraging words. And this is the only question that takes that aspect of information into consideration.

In conclusion, our analyses show that the GSES works reasonable well as a whole. Since the analyses are based on a large data set even relatively small evidence of misfit will appear to be statistically significant. There is clearly room for improvements of GSES. The targeting may for example be improved by adding more questions of medium difficulty. Also, further attention needs to be paid to the dimensionality of the GSES as well as to whether the psychometric properties of GSES are affected by using more recent data.

## Appendix 1

**Table 6:** The Swedish version of the General Self-Efficacy Scale, English wording in italics

		Tar helt avstånd *Not at all true*	Tar delvis avstånd *Hardly true*	Instämmer delvis *Moderately true*	Instämmer helt *Exactly true*
1	Jag lyckas alltid lösa svåra problem om jag bara anstränger mig tillräckligt. *I can always manage to solve difficult problems if I try hard enough.*				
2	Även om någon motarbetar mig hittar jag ändå utvägar att nå mina mål. *If someone opposes me, I can find the means and ways to get what I want.*				
3	Jag har inga svårigheter att hålla fast vid mina målsättningar och förverkliga mina mål. *It is easy for me to stick to my aims and accomplish my goals.*				
4	I oväntade situationer vet jag alltid hur jag skall agera. *I am confident that I could deal efficiently with unexpected events.*				
5	Till och med överraskande situationer tror jag mig klara av bra. *Thanks to my resourcefulness, I know how to handle unforeseen situations.*				
6	Tack vare min egen förmåga känner jag mig lugn även när jag ställs inför svårigheter. *I can remain calm when facing difficulties because I can rely on my coping abilities.*				
7	Vad som än händer klarar jag mig alltid. *I can solve most problems if I invest the necessary effort.*				
8	Vilket problem jag än ställs inför kan jag hitta en lösning. *When I am confronted with a problem, I can usually find several solutions.*				
9	Om jag ställs inför nya utmaningar vet jag hur jag skall ta mig an dem. *If I am in trouble, I can usually think of a solution.*				
10	När problem uppstår kan jag vanligtvis hantera dem av egen kraft. *I can usually handle whatever comes my way.*				
